# Comparative effectiveness of clozapine and non-clozapine atypical antipsychotics provided by the Brazilian National Health System in adults with schizophrenia

**DOI:** 10.3389/fpsyt.2024.1421501

**Published:** 2024-06-19

**Authors:** Júlio César Menezes Vieira, Edna Afonso Reis, Augusto Afonso Guerra, Helian Nunes de Oliveira, Cristina Mariano Ruas

**Affiliations:** ^1^ Program in Medications and Pharmaceutical Care – PPGMAF, School of Pharmacy, Federal University of Minas Gerais, Belo Horizonte, MG, Brazil; ^2^ Department of Statistics, Institute of Exact Sciences, Federal University of Minas Gerais, Belo Horizonte, MG, Brazil; ^3^ Brazilian National Health System’s Collaborating Centre for Technology Assessment and Excellence in Health (CCATES), School of Pharmacy, Federal University of Minas Gerais, Belo Horizonte, MG, Brazil; ^4^ Department of Social Pharmacy, School of Pharmacy, Federal University of Minas Gerais, Belo Horizonte, MG, Brazil; ^5^ Department of Social and Preventive Medicine, School of Medicine, Federal University of Minas Gerais, Belo Horizonte, MG, Brazil

**Keywords:** atypical antipsychotics, effectiveness, cohort study, Clozapine, Schizophrenia

## Abstract

**Introduction:**

Currently, 21 million people live with the disease, mostly in low to middle-income countries. We aimed to assess the survival of patients with schizophrenia using clozapine compared with non-clozapine atypical antipsychotics provided by the Brazilian National Health System using real-world data.

**Materials and methods:**

This is an open retrospective cohort study of patients diagnosed with schizophrenia to whom atypical antipsychotics were dispensed by the Brazilian National Health System between 2000 and 2015, based on deterministic-probabilistic pairing of administrative data records. The Kaplan-Meier method was used to estimate the cumulative probability of survival and the Cox proportional hazards model was adjusted to assess the risk factors for survival via the hazard ratio (HR).

**Result:**

Participants were 375,352 adults with schizophrenia, with an overall survival rate of 76.0% (95%CI 75.0–76.0) at the end of the cohort. Multivariate analysis indicated a greater risk of death for men (HR=1.30; 95%CI 1.27–1.32), older adults (HR=17.05; 95%CI 16.52–17.60), and in the Southeast region of Brazil (HR=1.20; 95%CI 1.17–1.23). Patients who used non-clozapine atypical antipsychotics had a 21% greater risk of death when compared to those taking clozapine (HR=1.21; 95%CI 1.14–1.29). Additionally, a history of hospitalization for pneumonia (HR=2.17; 95%CI 2.11–2.23) was the main clinical variable associated with increased risk of death, followed by hospitalization for lung cancer (HR=1.82; 95%CI 1.58–2.08), cardiovascular diseases (HR=1.44; 95%CI 1.40–1.49) and any type of neoplasia (HR=1.29; 95%CI 1.19–1.40).

**Discussion:**

This is the first published Brazilian cohort study that evaluated survival in people with schizophrenia, highlighting the impact of atypical antipsychotics. In this real-world analysis, the use of clozapine had a protective effect on survival when compared to olanzapine, risperidone, quetiapine, and ziprasidone.

## 1 Introduction

The lifetime prevalence of schizophrenia is approximately 0.3 to 1% of the general population, with an annual cost of US$ 150 billion in the United States (1, 2). Schizophrenia is one of the main diseases that causes loss of disability-adjusted life years (DALYs) (3, 4). Worldwide, 21 million people live with schizophrenia, mostly in low to middle-income countries (3). In the city of São Paulo, Brazil, the estimated prevalence of nonaffective psychoses is 1.9% (5).

Antipsychotics are the foundation of pharmacological treatment for schizophrenia and second-generation or atypical drugs are the recommended first-line treatment for schizophrenia due to their lower risk of extrapyramidal motor symptoms (6, 7). Atypical antipsychotics are associated with less risk of rehospitalization and treatment abandonment in patients with severe mental illness than their typical counterparts (8).

Although antipsychotics are effective in most patients with schizophrenia, about 30% of this population experiences few or no benefits (9). The antipsychotic clozapine is indicated for individuals with treatment-resistant schizophrenia (TRS) due to its superior efficacy in relation to other antipsychotics, with a clinical response of around 40 to 60% according to systematic reviews and meta-analyses on the topic (10–12).

Although clozapine is only recommended for people with TRS, it is linked to high quality of life scores (13) and significantly lower mortality rates due to natural causes and suicide when compared with other antipsychotics (14, 15). In a cohort of 2,370 people with TRS, all-cause mortality rates were higher in those who did not use clozapine and risk was twice as great in clozapine users after discontinuation (16).

Two retrospective cohort studies in Finland, with follow-up periods of 11 and 20 years, involving patients with schizophrenia, have demonstrated that clozapine significantly reduces overall mortality, cardiovascular mortality, and suicide mortality. Clozapine users had a lower cumulative mortality rate (15.6%) compared to those who did not use antipsychotics (46.2%). Additionally, clozapine exhibited the lowest risk of overall mortality compared to perphenazine, with an adjusted hazard ratio (HR) of 0.74 (17, 18).

The Brazilian National Health System (SUS in Portuguese) treats more than 190 million people and is a fundamental pillar of public health in the country. However, the disparity between demand for medications and effective access to them in the public health system requires critical analysis and a search for solutions. According to the Brazilian Institute of Geography and Statistics (19), 71.5% of the country’s population depend exclusively on the SUS for medical care, while only 30.5% are able to obtain at least one of their prescribed medications from the public system (19). The SUS provides the atypical antipsychotics risperidone, olanzapine, quetiapine, ziprasidone, and clozapine to people with schizophrenia free of charge (20).

Most studies have short follow-up periods, which compromises assessment of longitudinal effects, such as the effect of cumulative exposure to antipsychotics. Given that clozapine is similar to other antipsychotics in terms of effectiveness and safety; since there are no published studies on SUS patients who use antipsychotics that assess the risk of death stratified by the medication used, it is important to understand the factors associated with survival in patients with schizophrenia and discuss their repercussions on public policies. As such, this article aims to analyze survival in patients with schizophrenia who take atypical antipsychotics provided by the SUS and assess the associated social and clinical factors, using real-world data based on a 16-year follow-up.

## 2 Materials and methods

### 2.1 Study design and database

This is an open retrospective cohort study of patients diagnosed with schizophrenia to whom atypical antipsychotics were dispensed by the SUS between 2000 and 2015.

Brazil consists of 26 states and one Federal District, totaling 27 federative units. The Federal District, located in Brasília, is the seat of the federal government and does not belong to any state.

A person-centered national health database was created based on deterministic-probabilistic matching of the following administrative data records: The Outpatient Information System (SIA/SUS in Portuguese), Hospital Information System (SIH/SUS in Portuguese), and Mortality Information System (SIM in Portuguese). The construction and validation of this database were previously described and validated by Augusto Guerra and colleagues (21).

The SIA/SUS encompasses data on outpatient procedures and the provision of high-cost medications, processing all outpatient care production or high-cost medication dispensation. Meanwhile, the SIH/SUS processes all production related to hospital care, including discharges, transfers, medications, and deaths. The SIM data include the date and cause of death and secondary contributing causes coded by ICD-10 (21).

Inclusion criteria were individuals diagnosed according to the 10th revision of the International Statistical Classification of Diseases and Health-Related Problems (ICD-10) as having paranoid schizophrenia (F20.0), hebephrenic schizophrenia (F20.1), catatonic schizophrenia (F20.2), undifferentiated schizophrenia (F20.3), post-schizophrenic depression (F20.4), residual schizophrenia (F20.5), simple schizophrenia (F20.6), other schizophrenia (F20.8) or non-specific schizophrenia (F20. 9); as well as the date of the first recorded dispensation of an atypical antipsychotic in SIA and SIH registration between January 1, 2000 and December 31, 2014; and death recorded in SIM up to December 31, 2015.

Patients excluded from the cohort were those 17 years old or younger and 101 years or older, who used combinations of SUS-dispensed atypical antipsychotics, showed potential matching errors (duplicated records, with anomalous or incomplete drug dispensing numbers), event/time of less than 3 months in the cohort, three or fewer SIA outpatient visits, and treatment time less than or equal to 3 months (**Flow diagram 1**). The SUS provides a 3-month supply of atypical antipsychotics, after which time a new prescription must be requested from the attending physician. As such, a minimum of 3 months of SUS-dispensed antipsychotic use was established for participation in the cohort.

**Flow diagram 1:**
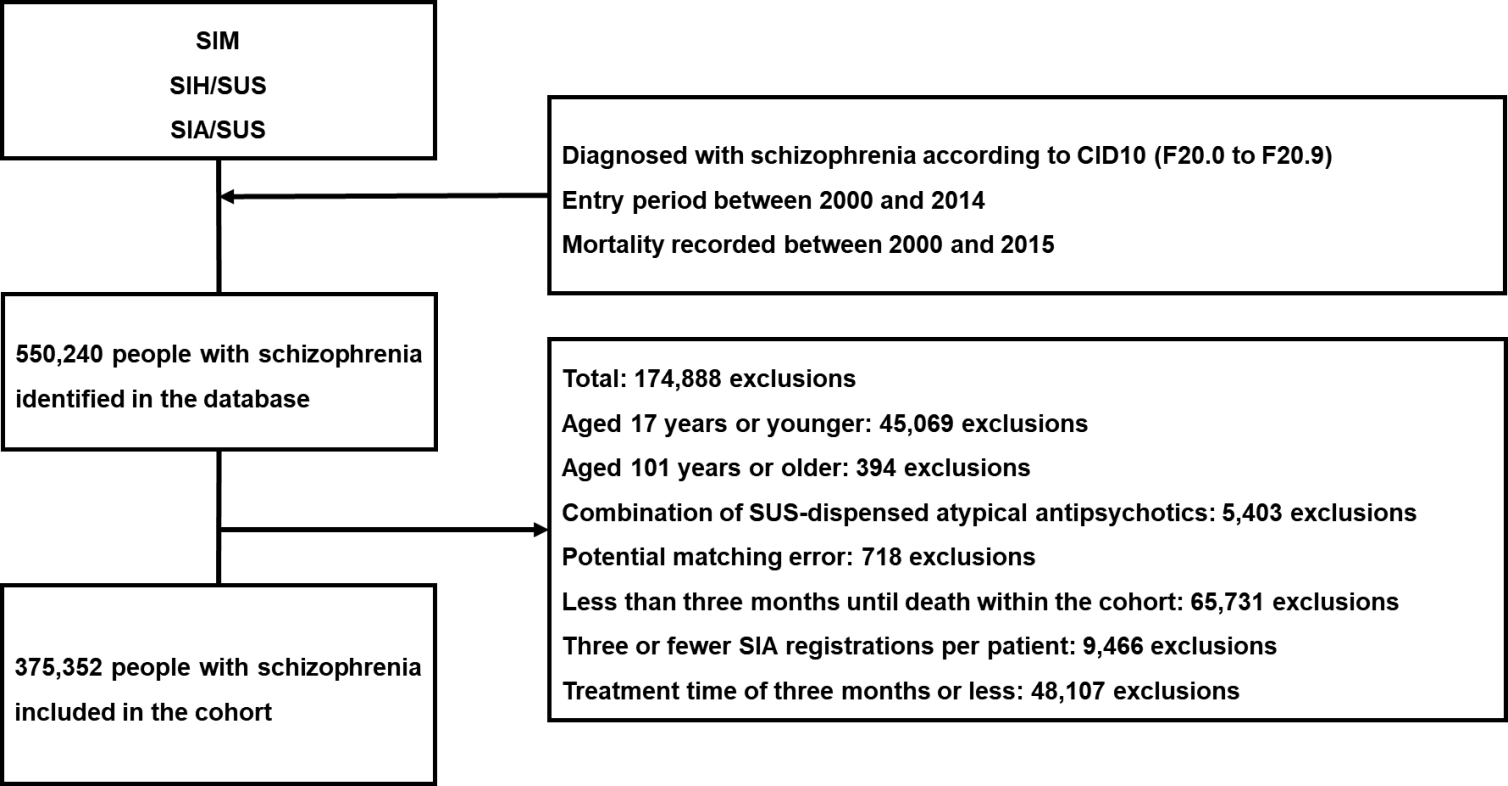
Selection of patients with schizophrenia who use atypical antipsychotics in a cohort. SUS, National Health System; SIM, Mortality Information System; SIH/SUS, Hospital Information System; SIA/SUS, Outpatient Information System.

Time until death in months was considered the dependent variable. Regarding the outcome “death within 16 years”, the groups were categorized as “survivors” and “deaths”. The independent variables were sex; age group (18–40 years, 41–59 years and ≥60 years); race/skin color (yellow, white indigenous, brown, black and not declared); region of residence in Brazil (North, South, Southeast, Northeast, and Center-West); atypical antipsychotics (risperidone, olanzapine, quetiapine, clozapine or ziprasidone) and hospitalization for cardiovascular disease (yes, no), pneumonia (yes, no), lung cancer (yes, no), and any neoplasia (yes, no).

### 2.2 Statistical analysis

The qualitative variables were described by absolute and relative frequencies and quantitative variables by measures of central tendency (mean and median) and variability (standard deviation-SD and interquartile range-IQR).

The association between each independent qualitative variable and the outcome “death within 16 years (yes or no)” was assessed using Pearson´s chi-squared test. The independent quantitative variables with normal distribution were compared between outcome groups via the Student’s t-test and those with non-normal distribution by the Wilcoxon rank or Kruskal-Wallis rank tests. Normality was analyzed using the Shapiro-Wilk test.

The Kaplan-Meier method was applied to estimate the cumulative probability of survival and the survival curves were compared by the log-rank test. The hazard ratio (HR) for the time until death and its 95% confidence interval (95%CI) were calculated by fusing the Cox proportional hazards model in both univariate and multivariate analysis. The variables with p<0.20 and clinical importance in the univariate model were included in the multivariate model. The final model contained variables with p<0.05. The goodness-of-fit of the multivariate model was analyzed based on Schoenfeld’s residuals. Data management and statistical analyses were performed using R software version 4.2.2. (R Foundation for Statistical Computing).

## 3 Results

### 3.1 Sociodemographic and clinical characteristics

Participants were 375,352 adults with schizophrenia, 334,955 (89.2%) of whom survived for the duration of the cohort, between 2000 and 2015. The mean and median overall survival time until death was 52.0 (SD=39.2) and 44.0 (IQR: 21.0–73.0) months, respectively.

The majority of the cohort was women (51.2%). The predominant age group was 18 to 40 years (46.1%), with a mean of 46 (SD=19) and median of 43 (IQR: 31–58) years. The predominant race/color was white (15.7%), but in 74.7% of cases, this information was not provided. The most prevalent region of residence was the Southeast (65.0%) and the most widely dispensed drug was risperidone (34.5%), followed by olanzapine (31.1%), quetiapine (22.6%), ziprasidone (6.3%) and clozapine (4.5%). During follow-up, the highest rate of hospitalization was for cardiovascular disease (3.4%), followed by pneumonia (3.1%), any neoplasia (0.7%), and lung cancer (0.1%) (**Table 1**).

**Table 1:** Sociodemographic and clinical characteristics of patients with schizophrenia using atypical antipsychotics provided by SUS/Brazil.

Characteristics		Overall n (%) 375 352 (100)	Survivors (%) 334 955 (89.2)	Death n (%) 40 397 (10.8)	p value
Sex	Female	192 264 (51.2)	170 562 (50.9)	21 702 (53.7)	<0.001
	Male	183 088 (48.8)	164 393 (49.1)	18 695 (46.3)	
Age group	18 - 40 years	172 987 (46.1)	167 945 (50.1)	5042 (12.5)	<0.001
	41 - 59 years	119 081 (31.7)	111 154 (33.2)	7927 (19.6)	
	Over 60 years	83 284 (22.2)	55 856 (16.7)	27 428 (67.9)	
Race/color	White	58 836 (15.7)	52 545 (15.69)	6291 (15.6)	<0.001
	Brown	19 952 (5.3)	18 410 (5.50)	1 542 (3.8)	
	Yellow	11 717 (3.1)	11 368 (3.39)	349 (0.9)	
	Black	4356 (1.2)	4001 (1.2)	355 (0.9)	
	Indigenous	47 (0.0)	46 (0.0)	1 (0.0)	
	ND	280 444 (74.7)	248 585 (74.2)	31 859 (78.9)	
Region of residence	Southeast	243 847 (65.0)	213 262 (63.7)	30 585 (75.7)	<0.001
	Northeast	61 258 (16.3)	56 807 (17.0)	4 451 (11.0)	
	South	37 495 (10.0)	34 558 (10.3)	2 937 (7.3)	
	Center-West	25 420 (6.7)	23 388 (7.0)	2032 (5.0)	
	North	7332 (2.0)	6940 (2.0)	392 (1.0)	
Atypical antipsychotics	Risperidone	129 436 (34.5)	113 377 (33.9)	16 059 (39.7)	<0.001
	Olanzapine	120 678 (32.1)	110 680 (33.0)	9 998 (24.7)	
	Quetiapine	84 751 (22.6)	72 889 (21.8)	11 862 (29.4)	
	Ziprasidone	23 673 (6.3)	22 276 (6.6)	1397 (3.5)	
	Clozapine	16 814 (4.5)	15 733 (4.7)	1081 (2.7)	
Hospitalization	Cardiovascular disease	12 690 (3.4)	8125 (2.4)	4565 (11.3)	<0.001
	Pneumonia	11 472 (3.1)	5553 (1.7)	5919 (14.7)	<0.001
	Lung cancer	455 (0.1)	248 (0.1)	207 (0.5)	<0.001
	Any neoplasia	2981 (0.8)	2367 (0.7)	614 (1.5)	<0.001

ND, not declared; SUS, National Health System.

### 3.2 Survival analysis

Across the 16-year cohort, 40,397 deaths (10.8%) occurred. Of those who died, most were older individuals (67.9%), women (53.7), white (15.6%), and resided in the Southeast (75.7%). Of these patients, 39.7% used risperidone, followed by quetiapine (29.4%), olanzapine (24.7%), ziprasidone (3.5%), and clozapine (2.7%). With respect to the clinical variables, 14.7% had been hospitalized for pneumonia, 11.3% for cardiovascular disease, 1.5% for any neoplasia, and 0.5% for lung cancer (**Table 1**).

**Graph 1:**
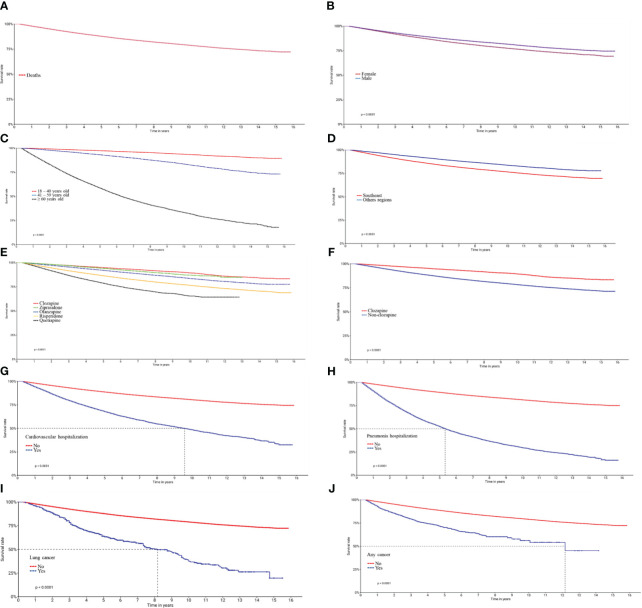
Kaplan-Meier curves for survival in patients with schizophrenia who use atypical antipsychotics provided by SUS/Brazil. **(A)** Overall survival of participants included in the cohort between 2000 and 2015; **(B)** Sex; **(C)** Age; **(D)** Region of residence; **(E)** Atypical antipsychotics; **(F)** Clozapine and non-clozapine antipsychotics; **(G)** Hospitalization for cardiovascular disease **(H)**, pneumonia **(I)**, any neoplasia **(J)** and lung cancer. Survival rates were estimated using the Kaplan-Meier method and compared via the log rank test.

At the end of the cohort, after 150 months (12.5 years), the overall survival rate of the individuals with schizophrenia was 76.0% (75.0%-76.0%CI). Women, older individuals, and those who lived in the Southeast had a significantly lower probability of survival, with almost half the older adults dying within 6.2 years (75 months). History of hospitalization for pneumonia was the main survival-shortening variable since almost half these patients had died within around 5.3 years (64 months) of the cohort; whereas hospitalization for cardiovascular disease, lung cancer, and any neoplasia were responsible for 50% of deaths in 9.5 years (114 months), 8.0 years (96 months) and 12.2 years (146 months), respectively (**Graph 1**).

In relation to atypical antipsychotics, patients with the longest mean/median time until death (in months) who used clozapine (68.8/55.0), followed by olanzapine (58.5/51.0), ziprasidone (55.9/52.0), risperidone, (53.3/44.0) and quetiapine (36.5/30.0) (**Table 2**).

**Table 2:** Estimated time until death and Kaplan Meier survival rate up to 150 months in patients with schizophrenia according to the atypical antipsychotic used - SUS/Brazil.

Atypical antipsychotic (n)		Average time until death (SD) (months)	Median time until death (Q1; Q3) (months)	Survival rate Kaplan Meier % (95%CI)
Clozapine	(n=16,814)	68.8 (49.1)	55.0 (31.0; 98.0)	85 (84.0–86.0)
Olanzapine	(n=120,678)	58.5 (42.0)	51.0 (24.0; 84.0)	80 (79.0- 80.0)
Ziprasidone	(n= 23,673)	55.9 (34.6)	52.0 (28.0; 80.0)	85 (83.0–86.0)
Risperidone	(n=129,436)	53.3 (40.2)	44.0 (22.0; 74.0)	72 (72.0–73.0)
Quetiapine	(n= 84,751)	36.5 (25.3)	30.0 (15.0; 53.0)	64.0 (63.0–66.0)
Overall	(n=375,352)	52.0 (39.2)	44.0 (21.0; 73.0)	76.0 (75.0–76.0)
p value		<0.001	<0.001	< 0.001

SD, standard deviation; Q, quartile; 95%CI, 95% confidence interval.

Survival rates among those who used clozapine and ziprasidone were 85.0 (95%CI 84.0–86.0) and 85.0% (95%CI 83.0–86.0), respectively, higher than olanzapine at 80.0% (95%CI 79.0–80.0), risperidone at 72.2% (95%CI 72.0–73.0) and quetiapine at 64.0% (95%CI 63.0–66.0) across 150 months (**Table 2**). The survival rate for clozapine was significantly higher when compared to the remaining antipsychotics in a single group (**Graph 1F**).

### 3.3 Univariate analysis

Univariate analysis in the Cox model indicated greater risk of death for women, older individuals, and those who live in the Southeast. Risk of death was almost six times higher for records of hospitalization for pneumonia (HR=5.68; 95%CI 5.52–5.84) during the cohort, surpassing hospitalization for cardiovascular disease (HR=3.26; 95%CI 3.16–3.36), lung cancer (HR=3.55; 95%CI 3.10–4.07) and any neoplasia (HR=2.89; 95%CI 2.67–3.13) (**Table 2**).

Risk of death was significantly greater in patients who used olanzapine (HR=1.52; 95%CI 1.44–1.62), quetiapine (HR=4.04; 95%CI 3.81–4.29), risperidone (HR=2.59; 95%CI 2.44–2.74) and ziprasidone (HR=1.12; 95%CI 1.04–1.21) when compared to clozapine during the 16-year follow-up. Risk of death for the grouped antipsychotics other than clozapine was twice as high as that of clozapine (HR=2.30; 95%CI 2.17- 2.43) (**Table 3**).

**Table 3:** Univariate analysis of the social and clinical characteristics of patients with schizophrenia who use atypical antipsychotics.

Characteristic		HR	95%CI	p value
Sex	Female	1	1	
	Male	0.82	0.81- 0.84	<0.001
Age group	18 - 40 years	1	1	
	41 - 59 years	2.64	2.55- 2.73	<0.001
	≥ 60 years	18.7	18.20- 19.30	<0.001
Region of residence	Southeast	1	1	
	Center-West	0.59	0.57- 0.62	<0.001
	Northeast	0.64	0.62- 0.66	<0.001
	North	0.45	0.41- 0.50	<0.001
	South	0.58	0.56- 0.60	<0.001
Atypical antipsychotic	Clozapine	1	1	
	Olanzapine	1.51	1.41- 1.60	<0.001
	Quetiapine	4.1	3.85- 4.37	<0.001
	Risperidone	2.48	2.33- 2.64	<0.001
	Ziprasidone	1.11	1.03- 1.20	0.009
Atypical antipsychotic	Clozapine	1	1	
	Non-clozapine	2.24	2.11- 2.38	<0.001
Hospitalized for cardiovascular disease	No	1	1	
	Yes	3.26	3.16- 3.36	<0.001
Hospitalized for pneumonia	No	1	1	
	Yes	5.68	5.52- 5.84	<0.001
Hospitalized for lung cancer	No	1	1	
	Yes	3.55	3.10- 4.07	<0.001
Hospitalized for any neoplasia	No	1	1	
	Yes	2.89	2.67- 3.13	<0.001

ND, Not declared; SUS, National Health System; HR, Hazard Ratio; 95%CI, 95% confidence interval.

### 3.4 Multivariate analysis

Multivariate analysis in the Cox model showed that, overall, there was a greater risk of death for men (HR=1.30; 95%CI 1.27–1.32), older adults (HR=17.05; 95%CI 16.52–17.60) and those who live in the Southeast (HR=1.20; 95%CI 1.17–1.23). A history of hospitalization for pneumonia (HR=2.17; 95%CI 2.11–2.23) was the main clinical risk factor for death along with lung cancer (HR=1.82; 95%CI 1.58–2.08), cardiovascular diseases (HR=1.44; 95%CI 1.40–1.49) and any type of neoplasia (HR=1.29; 95%CI 1.19–1.40). Additionally, patients who used non-clozapine antipsychotic had a 21% higher risk of death than those who received clozapine (HR=1.21; 95%CI 1.14–1.29) (**Table 4**).

**Table 4:** Multivariate analysis of the social and clinical characteristics of patients with schizophrenia who use atypical antipsychotics.

Characteristic		HR	95%CI	p value
Sex	Female	1		
	Male	1.30	1.27–1.32	<0.001
Age group	18 - 40 years	1		
	41 - 59 years	2.63	2.54–2.72	<0.001
	≥ 60 years	17.05	16.52–17.60	<0.001
Region of residence	Other regions	1		
	Southeast	1.20	1.17–1.23	<0.001
Atypical antipsychotic	Clozapine	1		<0.001
	Non-clozapine	1.21	1.14–1.29	
Hospitalized for cardiovascular disease	No	1		
	Yes	1.44	1.40–1.49	<0.001
Hospitalized for pneumonia	No	1		
	Yes	2.17	2.11–2.23	<0.001
Hospitalized for lung cancer	No	1		
	Yes	1.82	1.58–2.08	<0.001
Hospitalized for any neoplasia	No	1		
	Yes	1.29	1.19–1.40	<0.001

SUS, National Health System; HR, Hazard Ratio; 95%CI, 95% confidence interval.

However, Schoenfeld’s global testing resulted in p<0.05, demonstrating poor goodness-of-fit for the multivariate model due to violation of the proportional hazard assumption. Only in the Kaplan-Meier survival graphs was the proportional hazard assumption of the individual variables not violated over time (**Graph 1**).

## 4 Discussion

This study evaluated a large, open nationwide retrospective cohort of patients with schizophrenia and is an important source of evidence in investigating the factors associated with survival and long-term antipsychotic use, unprecedented in low and middle-income countries such as Brazil.

Multivariate analysis indicated greater risk of death for men, older adults, and those who lived in the Southeast over the 16-year follow-up. Most of the participants and data for this study originate from the Southeast region of Brazil. The states of Espírito Santo, Minas Gerais, Rio de Janeiro, and São Paulo constitute the Southeast region. As the economic center of the country, the Brazilian Southeast has the highest per capita income and population density. It is also where the largest cities, including São Paulo and Rio de Janeiro, are located, offering the finest urban infrastructure. This also induces internal migration within the country. Moreover, this regional disparity leads to inequity in access to health services, evidenced by the greater difficulty in accessing healthcare for individuals from the North and Northeast regions compared to those from the Southeast region. Therefore, the Southeast region possesses the largest health data banks in the country (22, 23).

The main clinical risk factor for death was hospitalization for pneumonia, which surpassed hospitalization for cardiovascular diseases. Previous research demonstrated that pneumonia is responsible for around half of all deaths in psychiatric hospitals, with a higher incidence among patients with schizophrenia than in the general population. In studies conducted in Taiwan and England, patients with schizophrenia were around twice as likely to die from pneumonia (24, 25). Atypical antipsychotics, particularly clozapine, are associated with an increased risk of pneumonia in people with schizophrenia, due to bronchoaspiration resulting from sialorrhea, a common adverse event (26–28).

Hospitalization for lung cancer was the second clinical variable linked to greater risk of death in people with schizophrenia, surpassing any neoplasia. Previous investigations reported increased risk of cancer in people with schizophrenia in comparison to the general population (29). In a systematic review of epidemiological studies, patients with schizophrenia diagnosed with cancer had a 40% higher risk of mortality, with the main cancers being breast, colon and lung (30, 31).

Hospitalization for cardiovascular disease was a significant risk factor in reducing survival. Studies also found an association between cardiovascular diseases and greater risk of death in patients with schizophrenia. In a review by Nielsen et al. (32), a quarter of individuals with schizophrenia had a shorter life expectancy due to cardiovascular diseases (32). In Sweden, a study of 46,911 people with schizophrenia treated in hospital between 1987 and 2010 observed a two to three-fold higher risk of mortality for cardiovascular and cerebrovascular diseases when compared with the general population (33).

Although antipsychotics have been linked to cardiovascular risk factors, a meta-analysis of 135 studies identified a lower risk of mortality for the use of antipsychotics when compared to not taking these drugs in individuals with schizophrenia, particularly for clozapine and extended-release atypical antipsychotics (14).

Dispensing clozapine to people with schizophrenia was a protective factor for survival when compared with non-clozapine atypical antipsychotics in this 16-year cohort. Other studies have reported similar findings. Cho et al. (34) conducted a 7-year cohort of 2,837 individuals with TRS and found that clozapine had a protective effect on all-cause mortality. After controlling for possible confounding factors, clozapine reduced the risk of all-cause mortality by 39% (34). Despite the risk of adverse cardiovascular effects attributed to clozapine, in a systematic review of 24 studies, mortality rates were 34% lower in patients under continuous clozapine treatment when compared with those who used other antipsychotics (35).

Although clozapine is clinically indicated for approximately 30% of patients with TRS, prescription rates were lower than expected for high-income countries and the use of nontherapeutic doses (36, 37). In our study, clozapine was only prescribed to 4.5% of participants, whereas Massuda et al. found that the clozapine prescription rate in Brazil increased from 7.2 to 10.9% between 2010 and 2020 (38). The causes of low clozapine indication include lack of training among attending physicians, fear of side effects such as agranulocytosis, and strict hematological monitoring (39).

However, there are concerns that long-term clozapine use may result in more adverse effects than a possible protective effect on survival. As such, a study of 62,250 patients with schizophrenia assessed the association between severe clinical morbidities and prolonged use of antipsychotics based on hospitalization data over a 20-year follow-up. The use of clozapine was associated with a lower risk of all-cause mortality, including cardiovascular disease and suicide (17). The results of the Schizophrenia Economics and Effectiveness Assessment (SCHEEA) indicated that Brazilian SUS patients with schizophrenia treated with clozapine obtained a higher average quality of life score, followed by olanzapine, risperidone, ziprasidone, and quetiapine (13).

Since clozapine is reserved for TRS, interpretation of the protective effect of clozapine presents an important bias because more severe patients are subject to greater clinical monitoring when using this antipsychotic. In order to understand the effect of intensive clinical monitoring on clozapine and mortality, 14,754 individuals with schizophrenia, schizoaffective, and bipolar disorders were monitored. The data obtained suggested that the protective effect of clozapine was maintained with no link to more careful clinical care, even after removing other potential confounders (40). The protective effect on survival in patients with schizophrenia is lost when clozapine is suspended and the risk of mortality is 2.50 times greater once it is discontinued (RR=2.50; 95%CI:1.50–4.17) (16).

As previously discussed, our findings align with those of studies conducted in other countries. Pneumonia hospitalization emerged as the primary risk factor for mortality in our investigation, corroborating results from studies in Taiwan and England. In Sweden, individuals with schizophrenia exhibit a two- to threefold increased risk of mortality from cardiovascular and cerebrovascular diseases compared to the general population, while our study found a 1.4-fold increased risk. Consistent with our discovery of clozapine’s protective role in individuals with schizophrenia, Cho et al.’s study (2014) conducted in South London also demonstrated a reduced mortality risk associated with clozapine use. Regarding Brazilian scientific literature, we identified no other studies with the same objective of evaluating clozapine’s effectiveness. Therefore, this is the first published Brazilian study that assesses the impact of atypical antipsychotics within the public healthcare system. While Brazil is the fifth highest country, the effectiveness result can reflect the reality of different countries worldwide.

Our study has several limitations. Since it is a retrospective cohort, the veracity of the data collected depends on correctly recording and inputting the information into the database. Approximately a third of Brazilians can obtain at least one of their prescribed medications through the public system (19). Limited access to medications in Brazil includes the unavailability of medications in health services, poor adherence, lack of education and resources, restricted knowledge about the disease, and social disparities (29.7% higher use among whites) (41, 42). The protective effect of clozapine on survival in this population should be assessed with caution since the drug is indicated for TRS. Additionally, this group of individuals receives more care when using clozapine due to the peculiarities of management and hematological control.

Furthermore, Schoenfeld global testing demonstrated poor goodness-of-fit for the multivariate model. However, it is important to note that violation of the proportional hazard assumption does not necessarily invalidate the analysis, but requires a more careful approach and correct result interpretation. The regression model produced a relative risk rate that does not quantify absolute effects or associations. Moreover, the combined effect of two or more independent variables on the risk of failure may not be simply the sum of their individual effects, but rather a complex interaction that varies over time.

Despite these limitations, the results obtained are sufficiently robust to add valuable information to understanding survival in people with schizophrenia in Brazil. The database consisted of 375,352 patients who represent the Brazilian population using atypical antipsychotics treated by the SUS. This large sample ensures greater reliability of the results in terms of the power of the hypothesis tests, which can minimize the potential bias errors caused by a small or rigorously selected sample. Finally, to the best of our knowledge, this is the first published Brazilian study to assess the survival of people with schizophrenia and the comparative impact of atypical antipsychotics.

## 5 Conclusion

In this Brazilian cohort, men with schizophrenia, over the age of 60 years, residing in the Southeast and with a history of hospitalization for pneumonia, cardiovascular disease, neoplasias, and lung cancer were at greater risk of death over a 16-year follow-up. The use of clozapine had a protective effect on death when compared to non-clozapine atypical antipsychotics in this real-world cohort. The results on the effectiveness of antipsychotics are crucial for guiding public policies and health actions that aim to update clinical guidelines and protocols for treating psychotic disorders like schizophrenia.

## Data availability statement

The datasets presented in this article are not readily available due to security requirements. They are stored on a secure web server at the Federal University of Minas Gerais, which meets strict standards for handling confidential research data. Requests to access the datasets should be directed to Prof. Augusto Afonso Guerra Jr. at augustoguerramg@gmail.com.

## Ethics statement

The studies involving humans were approved by Research Ethics Committee of the Federal University of Minas Gerais (CAAE 16334413.9.0000.5149) and all patient data remained confidential. The studies were conducted in accordance with the local legislation and institutional requirements. Written informed consent for participation was not required from the participants or the participants’ legal guardians/next of kin in accordance with the national legislation and institutional requirements.

## Author contributions

JV: Writing – review & editing, Writing – original draft. ER: Supervision, Writing – review & editing, Writing – original draft. AG: Writing – review & editing, Writing – original draft, Supervision. HO: Writing – review & editing, Writing – original draft, Supervision. CR: Writing – review & editing, Writing – original draft, Supervision.
